# Knockdown resistance (kdr)-associated organochlorine resistance in mosquito-borne diseases (*Culex pipiens*): A systematic review and meta-analysis

**DOI:** 10.1016/j.heliyon.2024.e41571

**Published:** 2024-12-30

**Authors:** Ebrahim Abbasi, Salman Daliri, Asghar Talbalaghi, Fatemeh Mehrpouya, Maryam Hasanzadeh arab, Atena Aslvaeli, Mohammad Djaefar Moemenbellah-Fard

**Affiliations:** a Research Center for Health Sciences, Institute of Health, Shiraz University of Medical Sciences, Shiraz, Iran; b Department of Medical Entomology and Vector Control, School of Health, Shiraz University of Medical Sciences, Shiraz, Iran; c Department of Medical Entomology, Faculty of Health, Ahvaz Jundishapur University of Medical Sciences, Ahvaz, Iran

**Keywords:** Knockdown resistance, Organochlorine insecticide, *Culex pipiens*, Systematic study of reviews and meta-analysis

## Abstract

**Background:**

*Culex pipiens* is the vector of a large number of pathogens in humans. Use of insecticides to deal with this vector is the most important way to controlling it. However, in recent decades, resistance to insecticides has been reported in this vector. One of the main insecticides used to fight this vector is organochlorine insecticides. Accordingly, this study was conducted to investigate the prevalence of Knockdown resistance (kdr) in *Culex pipiens* against organochlorine insecticides.

**Methods:**

This study was conducted via systematic review and meta-analysis approach in the field of kdr prevalence in *Culex pipiens* against organochlorine insecticides. Accordingly, during the search in the scientific databases PubMed, Web of Science, Biooan.org, Embase, ProQuest, Scopus, and Google Scholar without time limit until the end of November 2023, all related articles were extracted and analyzed. The statistical analysis of the data was performed using random and fixed effects model in the meta-analysis, Cochran's test, *I*^*2*^ index, and meta-regression by STATA software version 17.

**Results:**

seven studies with a sample size of 2029 *Culex pipiens* were included in the meta-analysis process. Based on the findings, the kdr resistance prevalence against Deltamethrin, Malathion, Permethrin, and DDT insecticides was estimated as 30.6 %, 42 %, 17.9 %, and 76.3 % respectively. Among them, the highest resistance was observed to DDT and the lowest to Permethrin.

**Conclusion:**

Based on the findings, a large proportion of *Culex pipiens* mosquitoes were resistant to DDT insecticide. However, this vector was highly sensitive to Deltamethrin, Malathion, and Permethrin insecticides. Given the different resistance ratios in different regions of the world, it is recommended to conduct studies on the prevalence of kdr in *Culex pipiens*.

## Highlights

• **Significance of *Culex pipiens***: This mosquito species is a major vector for several pathogens affecting humans, such as the West Nile virus, Rift Valley fever virus, and parasites causing filariasis*. Culex pipiens* thrives in urban environments due to human activities, making it a significant public health threat.
• **Insecticide Resistance**: The article emphasizes the growing resistance of *Culex pipiens* to insecticides, particularly organochlorines. This resistance, known as knockdown resistance (kdr), arises from mutations in the voltage-gated sodium channels (VGSC) in the mosquito's nervous system.
• **Meta-Analysis Findings**: The meta-analysis included seven studies, involving 2029 *Culex pipiens* mosquitoes across different regions. The estimated prevalence of kdr resistance against various insecticides is as follows: Deltamethrin: 30.6 %, Malathion: 42 %, Permethrin: 17.9 %, DDT: 76.3 % (highest resistance).
• **Geographical Variation**: Resistance ratios vary by region, with studies from countries like China, Iran, Morocco, Egypt, and the USA. For example, in Iran, *Culex pipiens* was highly resistant to DDT but sensitive to deltamethrin and malathion.
• **Mutations Leading to Resistance**: Two main mutations, L1014F and L1014S, in the VGSC gene, are identified as causes of kdr resistance in *Culex pipiens.* These mutations reduce the mosquito's sensitivity to insecticides, with the L1014F mutation playing a significant role in resistance to both organochlorine and organophosphate insecticides.
• **Importance of Sensitivity Studies**: Determining the sensitivity or resistance of mosquito populations to insecticides is crucial for effective control measures. The article calls for further research, especially in areas where this mosquito is endemic, to guide the use of appropriate and effective insecticides.
• **Conclusion**: The highest kdr resistance was observed against DDT, while *Culex pipiens* remained more sensitive to deltamethrin, malathion, and permethrin. Continuous monitoring and targeted research are recommended to assess and combat insecticide resistance in this important vector.

## Graphical abstract

**Figure:**
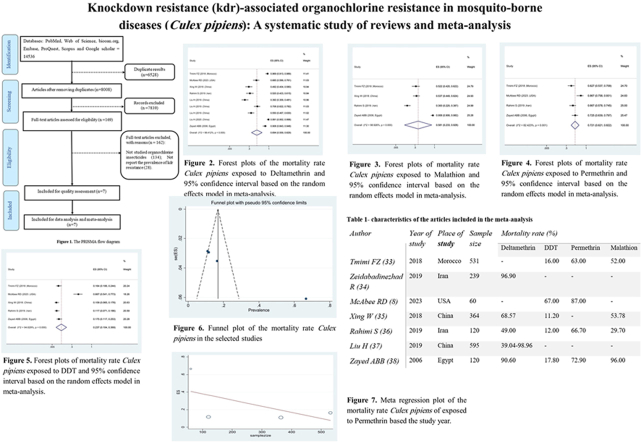

## Teaser

Author Summary: *Culex pipiens* is a significant vector for various human pathogens, and insecticides are crucial for its control. However, resistance to these insecticides, particularly organochlorines, has been increasingly reported. This study aimed to assess the prevalence of Knockdown resistance (kdr) in *Culex pipiens* against organochlorine insecticides through a systematic review and meta-analysis of existing studies. Data from seven studies involving 2029 *Culex pipiens* were analyzed, revealing kdr resistance rates of 30.6 % for Deltamethrin, 42 % for Malathion, 17.9 % for Permethrin, and 76.3 % for DDT, with DDT resistance being the highest. The findings emphasize the need for further studies on kdr prevalence in *Culex pipiens* across different regions.

## 1 Introduction

*Culex* is one of the most widespread mosquito species in the world [1]. They opportunistically feed on humans and animals, which provides suitable conditions for the transmission of common diseases between humans and animals and is a serious threat to public health [2]. *Culex* has adapted to human habitats over the years and is expanding in the urban environment due to the rapid and unplanned expansion of cities as well as the lack of suitable environmental conditions, the presence of stagnant water, drains, organically polluted places, and pits [3,4].

*Culex pipiens* is one of the most important groups of Culex, which includes six members of *Cx. pallens Coquillet, Cx. Quinquefasciatus Say, Cx. australicus Dobrotworsky & Drummond, Cx. molestus Forskell, Cx. pipiens Linneaus* and *Cx. Globocoxitus* is Dobrotworsky (*Culex pallens* and *Culex molestus* listed as subspecies of *Culex pipiens*, following ICZN guidelines) [5,6]. *Culex pipiens* is an important vector of a large number of pathogenic pathogens and parasites in the world. This mosquito is known as a vector of West Nile virus (WNV), Rift Valley fever virus (RVFV), *Wuchereria bancrofti* (Cobbold, 1877, the causative agent of filariasis), and Japanese and St. Louis encephalitis [[7], [8], [9]].

Diseases transmitted through vectors (mosquitoes) continue to affect the public health of humans. Due to the lack of vaccination to prevent vector-borne diseases, combating them is considered the best method of intervention. As a result, nowadays chemical insecticides are mainly used to control the vectors. Four groups of insecticides including organochlorines, organophosphates, carbamates, and pyrethroids are the main insecticides used to fighting vectors [10]. Pyrethroids account for about 15 % of the insecticides used to combat vectors in the world. Pyrethroids are widely used to control vectors due to their low toxicity to humans and high lethal effect on insects [11,12].

Organochlorines, historically used to control vectors, act on the central and peripheral nervous systems of insects. They interact with voltage-gated sodium channels, leading to paralysis and death by inhibiting inactivation processes and increasing sensitivity to depolarization [13,14].

In response to the long-term, extensive, and incomplete use of these insecticides over time, it has led to the natural selection of insects and a reduction in the sensitivity of the target site to them, which is known as "kdr". It is caused by the insensitivity of the target site due to the mutation in the voltage-sensitive sodium channel gene (Vssc) [15]. Resistance to DDT and deltamethrin is often associated with mutations in the sodium channel gene, which reduces neuronal sensitivity to these insecticides [16].

In the studies conducted worldwide, kdr resistance was reported in *Culex pipiens* and it was shown that the ratio of resistance is different in different countries [17]. Studies have mentioned that three groups of glutathione-S-trans-ferases (GST), esterase, and cytochrome P450 oxidases play a role in creating metabolic resistance to organochlorine, organophosphate, and pyrethroids in *Culex pipiens* [18]. In the field of Kdr resistance in *Culex pipiens*, it has been reported that two mutations L1014F and L1014S cause Kdr resistance in it [19].

Identifying mutations related to resistance is essential for managing together with using appropriate and effective insecticides to control insects. Note that *Culex pipiens* is a carrier of some pathogenic pathogens for humans and is now spreading in the world [20,21]. Determining the level of sensitivity or resistance to insecticides is essential to dealing with this vector. Accordingly, the present study was conducted to investigate the prevalence of kdr resistance in *Culex pipiens* against organochlorine insecticides via a systematic review and meta-analysis [22,23]. These findings suggest that regional differences in resistance could be influenced by variations in insecticide use practices, environmental factors, and genetic adaptations of mosquito populations. Further studies should explore these factors to guide targeted control strategies.

## 2 Materials and methods

This study was performed via systematic review and meta-analysis based on the guidelines of the Preferred Reporting Items for Systematic Reviews and Meta-Analyses (PRISMA) in the field of kdr prevalence in *Culex pipiens* against organochlorine insecticides [24]. This research has been registered in the International Prospective Register of Systematic Review (PROSPERO) under the code CRD42021231605.

### 2.1 Search strategy

The two researchers extract papers from scientific databases of PubMed, Web of Science, Biooan.org, Embase, ProQuest, Scopus, and Google Scholar using keywords Resistance, knockdown resistance, kdr, insecticide, Organochlorine insecticide, chlorinated insecticide, chlorophenyl, dichloroethane, DDT, parachlorophenyl, dichlorodiphenyldichloroethane, dieldrin, permethrin, deltamethrin, malathion, Culex and *Culex pipiens*. They then investigated the title, abstract and full text of the articles singularly and in combination using OR, AND and NOT operators without time limit until the end of November 2023.

### 2.2 Inclusion and exclusion criteria

Based on the PICO model, published English-language articles conducted on *Culex pipiens* as well as examining kdr resistance to organochlorine insecticides, and the prevalence of resistance or mortality in exposure to organochlorine insecticides as well as showing good quality were included in the study. The articles that were conducted on other insects, and other insecticides (except organochlorine insecticides), those not dealing with kdr resistance, lacking the desired quality, and in the review method, case report or Letters to the editor were excluded from the study.

### 2.3 Quality assessment

The quality assessment of the articles was done based on 22 parts of the STROBE (Strengthening the Reporting of Observational Studies in Epidemiology) checklist, which investigated compliance with the principles of writing and implementation in the title, the method of reporting findings, limitations, and conclusions. Each part of this checklist is given a score based on its importance; the maximum possible score is 33. Based on the obtained score, the studies were divided into three levels low, medium, and high quality [25].

### 2.4 Data extraction

Initially, the articles were investigated by two researchers independently by examining the title and abstract, taking into account the inclusion and exclusion criteria. Then, the full text of the articles was inspected by these researchers, and if the articles were rejected by two researchers, the reason was mentioned. Also, in case of disagreement between them, the article was refereed by a third researcher. Data extraction was performed using a pre-prepared checklist which included the first author's name, study place, study year, sample size, insecticide type, kdr resistance prevalence, and mortality.

### 2.5 Selection of studies

**Fig. 1:**
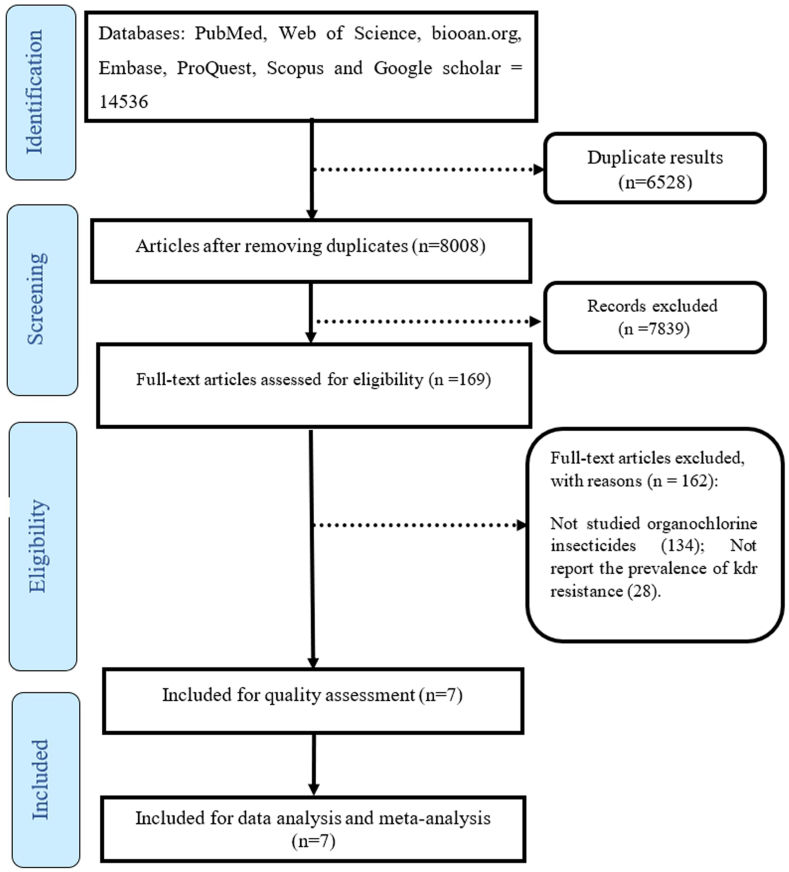
The PRISMA flow diagram.

A total of 14536 studies were extracted by searching the databases. At first, the articles were entered into the Endnote software, and after the initial review, 6528 articles were excluded from the study due to being duplicates. Next, by checking the titles and abstract of the articles, 7839 articles were removed as they were not relevant, and after reviewing the full text of the articles, 162 articles were removed due to lack of investigation of the prevalence of kdr resistance or resistance to organochlorine insecticide. Finally, 7 articles met the inclusion criteria and included in the meta-analysis process (Fig. 1).

### 2.6 Statistical analysis

Statistical analysis of the data was done using random and fixed effects models in meta-analysis, plus *I*^*2*^ index and Cochran's test. Further, funnel plot was used to investigate the publication bias and Meta regression to explore the relationship between the sample size and the prevalence of kdr. Data analysis was done using STATA software version 17.

## 3 Results

### 3.1 Overview of included studies and sample sizes

Seven studies with a sample size of 2029 *Culex pipiens* mosquitoes that were conducted between 2006 and 2023 were included in the study. Two studies were conducted in China, two in Iran, and one study each in Morocco, Egypt, and the United States. The characteristics of the reviewed articles are presented in Table 1.

**Table 1:** Characteristics of the articles included in the meta-analysis.

Author	Year of study	Place of study	Sample size	Mortality rate (%)			
				Deltamethrin	DDT	Permethrin	Malathion
Tmimi FZ [36]	2018	Morocco	531	–	16.00	63.00	52.00
Zeidabadinezhad R [37]	2019	Iran	239	96.90	–	–	–
McAbee RD [8]	2023	USA	60	–	67.00	87.00	–
Xing W [38]	2018	China	364	68.57	11.20	–	53.78
Rahimi S [39]	2019	Iran	120	49.00	12.00	66.70	29.70
Liu H [40]	2019	China	595	39.04–98.96	–	–	–
Zayed ABB [41]	2006	Egypt	120	90.60	17.80	72.90	96.00

**Fig. 2:**
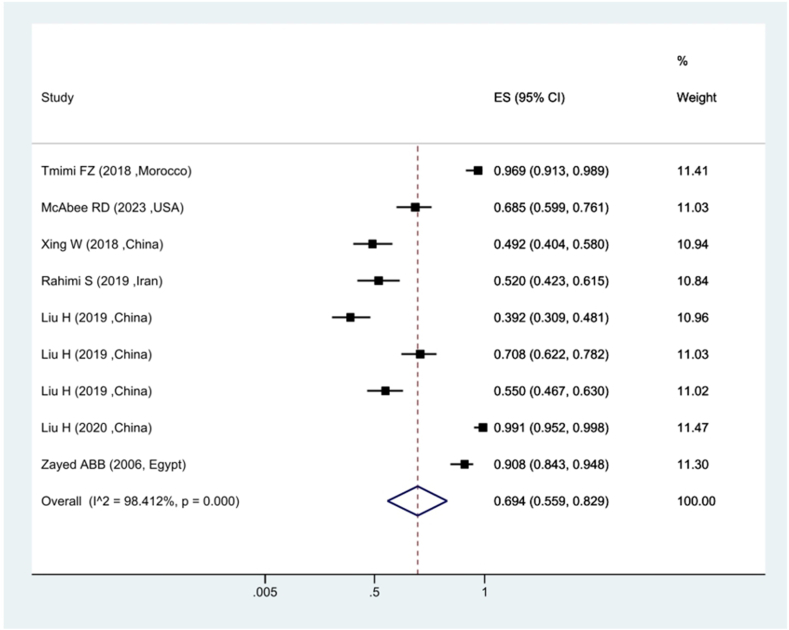
Forest plots of the mortality rate *Culex pipiens* exposed to Deltamethrin and 95 % confidence interval based on the random effects model in meta-analysis.

**Fig. 3:**
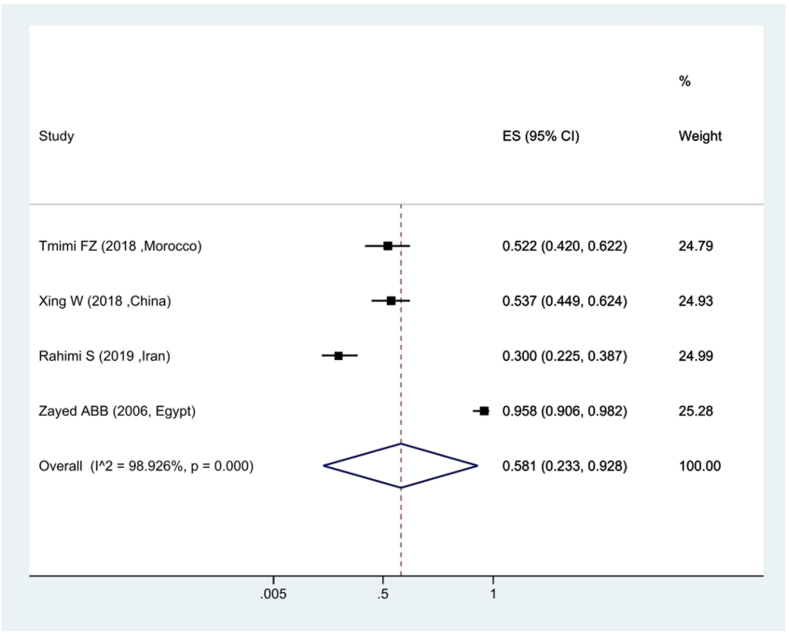
Forest plots of mortality rate *Culex pipiens* exposed to Malathion and 95 % confidence interval based on the random effects model in meta-analysis.

Based on the findings of the meta-analysis of the seven studies conducted on the prevalence of kdr resistance in *Culex pipiens* against Deltamethrin insecticide, the ratio of sensitivity to Deltamethrin insecticide was estimated at 69.4 %. showing 30.6 % of *Culex pipiens* mosquitoes were resistant to Deltamethrin. In terms of heterogeneity between studies, the *I*^*2*^ index was estimated at 98.41 %, indicating existence of heterogeneity between studies (Fig. 2). The investigation of the prevalence of *Culex pipiens* resistance against Malathion insecticide revealed that the prevalence of sensitivity in *Culex pipiens* mosquitoes was 58.1 %, demonstrating that about 42 % of *Culex pipiens* have kdr resistance to Malathion (Fig. 3).

### 3.2 Resistance rates for each insecticide

**Fig. 4:**
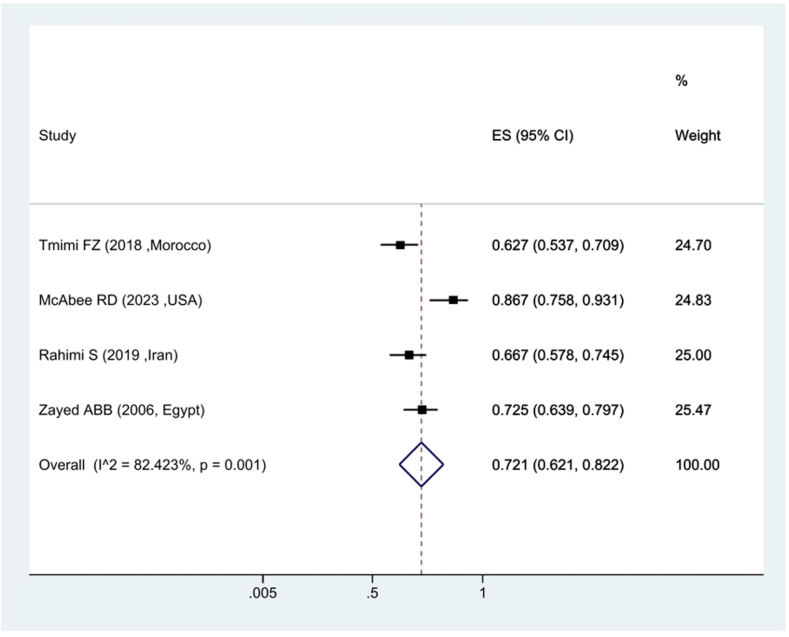
Forest plots of mortality rate *Culex pipiens* exposed to Permethrin and 95 % confidence interval based on the random effects model in meta-analysis.

**Fig. 5:**
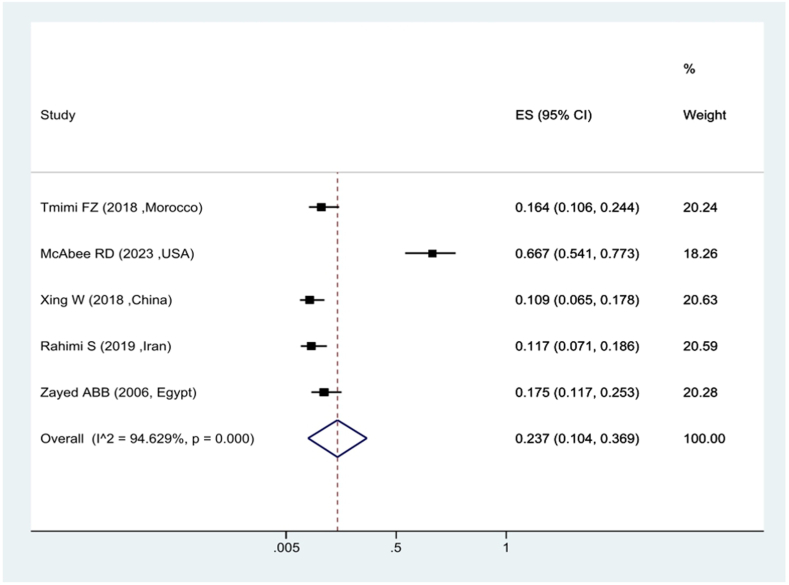
Forest plots of mortality rate *Culex pipiens* exposed to DDT and 95 % confidence interval based on the random effects model in meta-analysis.

In the context of the prevalence of kdr resistance in *Culex pipiens* against Permethrin insecticide, the findings revealed that 72.1 % of *Culex pipiens* were sensitive to Permethrin and it shows that 17.9 % of them had kdr resistance (Fig. 4). The attenuation ratio against DDT insecticide was estimated at 23.7 %, which shows that 76.3 % of *Culex pipiens* have kdr resistance against DDT (Fig. 5).

### 3.3 Heterogeneity and meta-regression findings

**Fig. 6:**
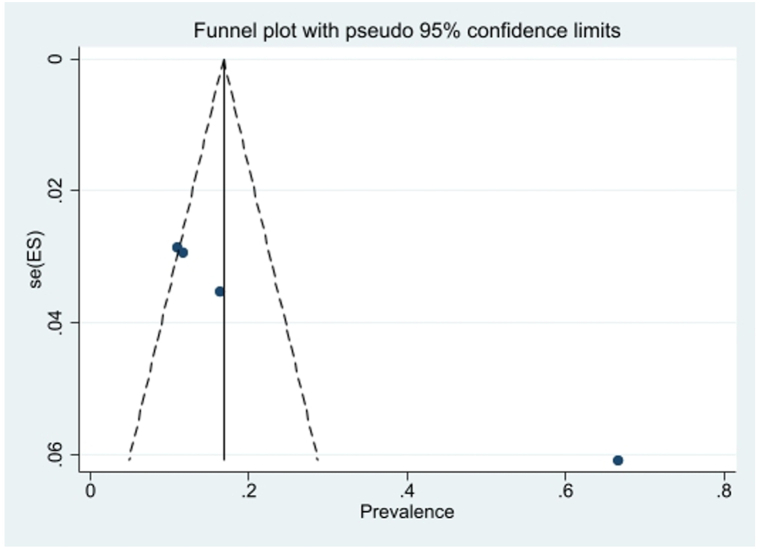
Funnel plot of the mortality rate *Culex pipiens* in the selected studies.

**Fig. 7:**
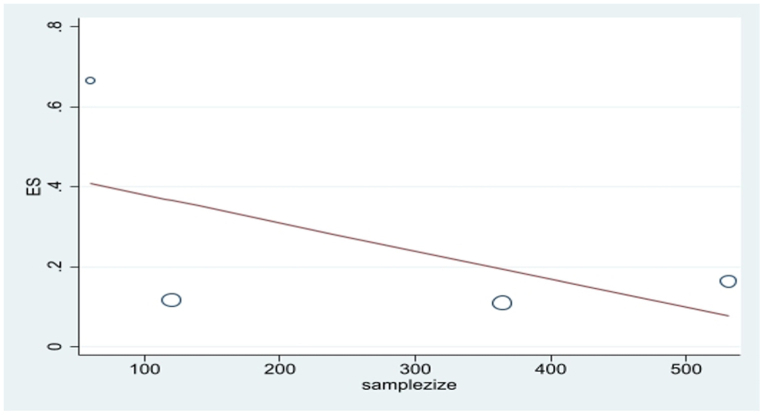
Meta regression plot of the mortality rate *Culex pipiens* of exposed to Permethrin based the study year.

The publication bias was examined using the funnel plot and Egger's test. Due to the symmetry of the funnel plot, it can be mentioned that publication bias did not occur, and the result of Egger's test was not significant in this regard (P = 0.14) (Fig. 6). Also, using meta-regression, the relationship between sample size and mortality ratio was investigated. According to the slope of the graph, the mortality ratio diminished with the increase of the sample size, showing the resistance ratio was higher in larger populations (Fig. 7).

Therefore, the meta-analysis included seven studies, with a total of 2029 *Culex pipiens* mosquitoes. The prevalence of resistance to DDT was the highest at 76.3 %, followed by Malathion (42 %), Deltamethrin (30.6 %), and Permethrin (17.9 %). Heterogeneity across studies was significant, indicating regional variability in resistance patterns.

## 4 Discussion

This study was conducted on the prevalence of kdr resistance in *Culex pipiens* against Deltamethrin, Permethrin, Malathion, and DDT insecticides via meta-analysis method. Based on the findings, the kdr resistance ratio against Deltamethrin, Malathion, Permethrin, and DDT insecticides was estimated as 30.6 %, 42 %, 17.9 %, and 76.3 % respectively. Among them, the highest resistance was observed to DDT and the lowest to Permethrin. It can be mentioned that the most effective insecticide to deal with *Culex pipiens* is Permethrin and Deltamethrin. In studies of insecticide target sites in *Culex pipiens*, G119S ace-1 and L1014F kdr mutants. Regarding resistance to DDT, these mutations were identified and shown to play a role in creating resistance in *Culex pipiens* [26,27].

Studies have also shown that the L1014F kdr mutation is widely present in *Culex pipiens*. This mutation plays an important role in resistance to organochlorine and organophosphate insecticides [28]. The mutation from leucine to serine (TTA to TCA) is another mutation identified in *Culex pipiens* resistance, which has been observed in different countries including China, America, and Japan [[29], [30], [31]]. The L1014C mutation with the substitution of leucine (TTA) for cysteine (TGT) is another mutation identified in *Culex pipiens* [29].

Resistance to insecticides like DDT in *Culex pipiens* poses significant public health risks, as it can undermine vector control programs and increase the spread of diseases such as West Nile virus and filariasis. Alternative control measures, such as the use of insecticides with different modes of action and integrated vector management (IVM), should be prioritized. Mosquitoes with the Phe/kdr mutation also showed high resistance to pyrethroids and DDT, but the Ser/kdr mutation confers high resistance to DDT and low resistance to pyrethroids [32]. The presence of various mutations in *Culex pipiens* indicates the presence of high resistance and their spread in different regions of the world. Because under selection pressure, carriers can acquire resistance and transfer it to the next generations, as a result, this phenomenon can lead to an increase in the prevalence of kdr resistance in *Culex pipiens* mosquitoes in the world. In a study conducted in northern Iran, the sensitivity ratio of *Culex pipiens* mosquitoes to DDT was low, but a large ratio of them was sensitive to deltamethrin [33].

In another study by Salim-Abadi et al. (2016) in Iran, *Culex pipiens* were sensitive to Deltamethrin and resistant to DDT [34]. In the study of Akiner et al. (2009) in Turkey, *Culex pipiens* was highly sensitive to Malathion, Deltamethrin, and Permethrin insecticides, but highly resistant to DDT [35].

In general, based on the findings of the present study, it can be mentioned that *Culex pipiens* is highly sensitive to Deltamethrin, Malathion, and Permethrin insecticides, but it has high kdr resistance to DDT insecticide. Accordingly, it is recommended to use effective insecticides to fight and control this vector. Also, due to the different resistance ratios in different regions of the world, it is recommended to conduct studies on the prevalence ratio of kdr resistance.

## 5 Conclusion

According to the findings, a large proportion of *Culex pipiens* mosquitoes were resistant to DDT insecticide. However, this vector was highly sensitive to Deltamethrin, Malathion, and Permethrin insecticides. Since few studies have been done in this field, it is recommended to conduct studies to evaluate the prevalence of resistance in countries where this vector is endemic.

## CRediT authorship contribution statement

**Ebrahim Abbasi:** Writing – review & editing, Writing – original draft, Validation, Software, Methodology, Formal analysis, Data curation, Conceptualization. **Salman Daliri:** Data curation, Conceptualization. **Asghar Talbalaghi:** Formal analysis, Data curation. **Fatemeh Mehrpouya:** Funding acquisition, Formal analysis. **Maryam Hasanzadeh arab:** Methodology, Investigation. **Atena Aslvaeli:** Project administration, Methodology. **Mohammad Djaefar Moemenbellah-Fard:** Supervision, Software.

## Ethics approval and consent to participate

Not applicable.

## Consent for publication

All authors declare their consent for publication.

## Data availability statement

All the data obtained from this study are included in the text of the article.

## Funding

This study received no grants from commercial, public, or nonprofit entities.

## Declaration of competing interest

The authors declare that they have no known competing financial interests or personal relationships that could have appeared to influence the work reported in this paper.
